# Antitumor potential of polyamines in cancer

**DOI:** 10.3724/abbs.2025030

**Published:** 2025-03-18

**Authors:** He Liu, Yi Liu, Xinyue Wang, Zhiwen Xiao, Quanxing Ni, Xianjun Yu, Guopei Luo

**Affiliations:** 1 Department of Pancreatic Surgery Fudan University Shanghai Cancer Center Shanghai 200032 China; 2 Department of Oncology Shanghai Medical College Fudan University Shanghai 200032 China; 3 Shanghai Pancreatic Cancer Institute Shanghai 200032 China; 4 Shanghai Key Laboratory of Precision Medicine for Pancreatic Cancer Shanghai 200032 China; 5 Pancreatic Cancer Institute Fudan University Shanghai 200032 China

**Keywords:** polyamines, cancer, oncogenic, signaling pathway

## Abstract

The dysregulation of polyamines in tumors has made polyamine metabolism an appealing target for cancer therapy. Gene mutations drive the reprogramming of polyamine metabolism in tumors, presenting promising opportunities for clinical treatment. The proposed strategies involve inhibiting polyamine biosynthesis while also targeting the polyamine transport system as antitumor approaches. A growing number of drugs aimed at polyamine biosynthesis and transport systems are undergoing clinical trials. Polyamine metabolism plays a role in regulating cancer signaling pathways, suggesting potential combination therapies for cancer treatment. Furthermore, supplemental polyamine substances have demonstrated antitumor activity, indicating that combining polyamines with downstream targets or immunotherapy could offer significant clinical benefits. These discoveries open new avenues for leveraging polyamine metabolism in anticancer therapy.

## Introduction

Polyamines are natural components in cells and body fluids and belong to a class of long-chain aliphatic compounds containing two or more amino groups. Important polyamines in mammals, such as putrescine, spermidine, and spermine, play crucial physiological roles. With multiple positive charges, polyamines exhibit strong affinity for nucleic acids and play a significant role in regulating processes such as replication, transcription, and cell division [1]. Polyamines are widely distributed in various animals, plants, and bacteria and play crucial roles in regulating cellular physiological functions such as cell proliferation and differentiation [2]. Furthermore, polyamines are also implicated in immune cell differentiation and the modulation of inflammatory responses [3]. Over the years, researchers have reported that polyamines are able to modulate ion channels and participate in transcriptional, translational and post-translational activities [ 4, 5]. Additionally, polyamines are involved in regulating chromatin remodeling, eukaryotic translation initiation factor 5A (eIF-5A) hypnosis and apoptosis [6]. Intracellular polyamines are strictly regulated at millimolar concentrations through the coordination of biosynthesis, degradation metabolism, and extracellular uptake transport in a dynamic equilibrium process [ 7, 8]. Extracellular polyamines are derived mainly from the diet, microbiota, and shed or damaged cells, where they are absorbed into the circulation or enter the cells to exert their functions [ 8, 9]. Intracellular polyamine biosynthesis decreases with age, and supplementation with spermine and spermidine has been shown to prolong lifespan by inhibiting inflammatory responses and promoting cellular autophagy [10].

Dysregulation of polyamine metabolism is commonly observed in various tumors and is directly associated with the initiation and progression of cancer [11]. Studies have demonstrated that compared with normal cells, tumor cells exhibit elevated levels of polyamines [ 12, 13]. Tumor cell proliferation requires an intracellular pool of polyamines for maintenance. Reducing intracellular polyamine levels in the body to enhance antitumor immune response is considered an effective antitumor strategy [14]. In addition, polyamine metabolism is interconnected with the microbiota and diet, contributing to the establishment of a tumor microenvironment that promotes cancer initiation and progression [8]. Some gene mutations and signaling pathways are involved in the regulation of polyamine metabolism disorders in tumor cells, providing rational targets for intervention in cancer therapy. In addition, recent evidence has shown that polyamines can enhance the body’s antitumor immune response, providing new evidence for the use of polyamines in cancer therapy. Therefore, new strategies for combination therapy utilizing polyamines should be explored for the treatment and prevention of multiple types of cancers.

Polyamine metabolism plays a crucial role in cell growth and proliferation, and its dysregulation in tumors provides new targets for cancer therapy. Genetic mutations and loss are the main factors driving the reprogramming of polyamine metabolism in tumors, which not only alter the pathways of polyamine synthesis and degradation but also affect the transport and distribution of polyamines within cells, suggesting new opportunities for clinical treatment. Currently, anticancer strategies targeting polyamine metabolism mainly include inhibiting polyamine biosynthesis and targeting the polyamine transport system. Numerous drugs are undergoing clinical trials to assess their effects on polyamine biosynthesis and transport systems in hopes of finding more effective cancer treatment methods. In addition, polyamine metabolism is involved in regulating cancer signaling pathways, suggesting that we can enhance therapeutic effects by combining polyamine metabolism inhibitors with other anticancer drugs or therapies through combination treatment. Notably, some polyamine supplements have also shown antitumor activity, indicating that combining polyamines with downstream targets or immunotherapy may result in significant clinical benefits. These findings pave new avenues for the use of polyamine metabolism in anticancer treatment, but further in-depth research is needed to explore its specific mechanisms and optimal clinical application strategies to overcome potential challenges and maximize its therapeutic potential.

In this review, we present several important oncogenic signaling pathways that contribute to dysregulated polyamine metabolism in tumors, as well as recent advances in specific new therapeutic strategies to control and prevent tumors using polyamine metabolism. In addition, we present new findings on the activation of host immunity by polyamine supplementation, providing promising targets and strategies for antitumor therapy.

## Polyamine Metabolism and Transport

### Polyamine biosynthesis and catabolism

**Figure 1:**
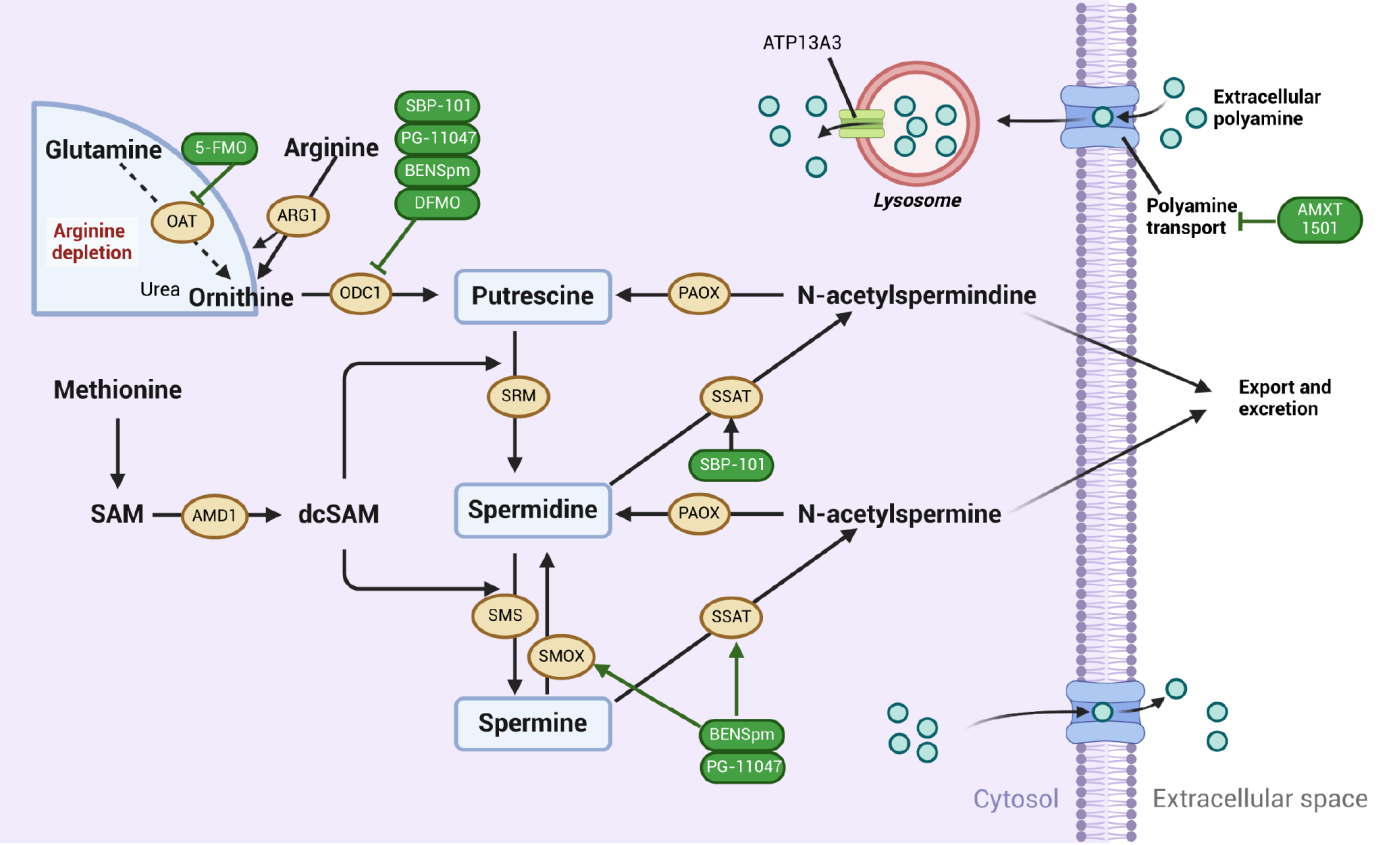
Figure 1 Polyamine biosynthesis and catabolism in cancer cells Major pathways and enzymes of polyamine metabolism in cancer cells. The process of polyamine formation in tumor cells includes uptake, biosynthesis, catabolism, and transporter transport. OAT, ornithine aminotransferase; AMD1, S-adenosylmethionine decarboxylase proenzyme; ARG1, arginase-1; ODC1, ornithine decarboxylase; dcSAM, decarboxylated S-adenosylmethionine; PAOX, peroxisomal N(1)-acetyl-spermine/spermidine oxidase; SAM, S-adenosylmethionine; SMOX, spermine oxidase; SSAT, spermidine/spermine N(1)-acetyltransferase 1; SMS, spermine synthase; SRM, spermidine synthase (Created in BioRender. Liu, H. (2025) https://BioRender.com/166g716).

Polyamine homeostasis is precisely controlled by the synergistic actions of their biosynthesis, catabolism, and transport processes ( Figure 1). Arginine is one of the precursor amino acids for polyamine biosynthesis in most organisms, and arginase 1 (ARG1) converts the amino acid arginine into ornithine [15]. Recent studies have shown that glutamine plays a role in polyamine biosynthesis in pancreatic cancer cells, particularly in the absence of arginine in the tumor microenvironment [16]. Ornithine is a precursor for polyamine biosynthesis, which is converted to putrescine by ornithine decarboxylase (ODC) and metabolized to spermidine and spermine by spermidine synthase (SRM) and spermine synthase (SMS) [ 1, 17]. ODC is regulated by many growth factors, oncogenes and tumor promoters and free of polyamine, and these factors may occur at the level of transcription, translation, and protein degradation [18]. The aminopropyl donor for each putrescine- or spermidine-producing spermine is decarboxylated S-adenosylmethionine (dcSAM), and the process is regulated by S-adenosylmethionine decarboxylase proenzyme (AMD1). These reactions are efficient and irreversible, and the catabolic pathway of polyamines can directly convert spermine to spermidine through the action of spermine oxidase (SMOX). Furthermore, with the involvement of the acetyl-CoA cofactor, the levels of polyamines can be modulated by spermidine/spermine N(1)-acetyltransferase 1 (SSAT) and peroxisomal N(1)-acetyl-spermine/spermidine oxidase (PAOX) [19]. The polyamine pool is regulated by biosynthetic and catabolic pathways to prevent the accumulation of excessive polyamines within cells.

### Polyamine transport system

The polyamine transport system (PTS) for the entry and exit of polyamines into and out of cells helps regulate the absorption of extracellular polyamines and the overall polyamine content within the organism. Mammalian PTSs are saturable, highly energy-dependent, and exhibit a high affinity for their substrates [20]. Free polyamines can enter cells via polyamine permeation or receptor-mediated endocytosis, as recent studies have shown [ 21, 22]. Upon entry into the cell, polyamines are translocated to the cytoplasm via polyamine-sequestering vesicles [20]. The late endolysosomal transporter ATP13A2 (PARK9) functions as a polyamine exporter, displaying a high affinity for spermine [23]. ATP13A2 promotes endocytosis by hydrolyzing ATP to transport polyamines into the cytoplasm, thereby affecting intracellular polyamine levels. Additionally, ATP13A3 has been identified as a key component of the mammalian polyamine transport system but is highly active in early and recycling endosomes for treatment and containment [24]. The polyamine transporter ATP13A3 has been shown to play a role in polyamine transport and in predicting the response to polyamine-targeted therapy in pancreatic cancer cells [25]. As a novel polyamine transporter, ATP13A4 was also found to potentially play an important role in increased polyamine uptake in breast cancer cells [26]. Spermidine and spermine are stored in synaptic vesicles or synapse-like microvesicles in astrocytes through active transport mediated by the solute carrier 18B1 (SLC18B1) and are released into the cytoplasm through exocytosis upon stimulation [27].

## Polyamines and Cancers

### Oncogenes and tumor suppressor genes involved in polyamine metabolism

**Figure 2:**
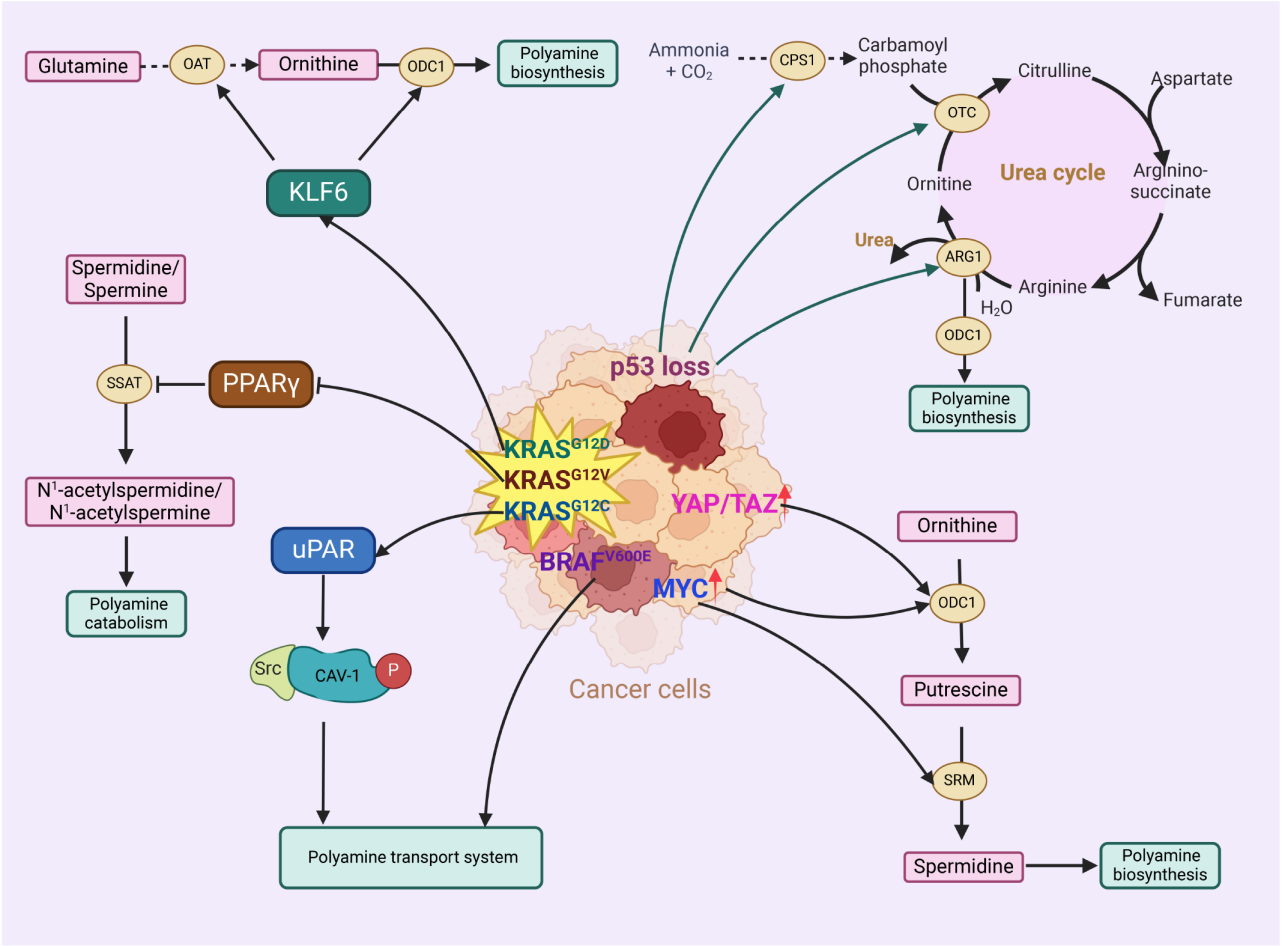
Figure 2 Oncogenes and tumor suppressor genes affect polyamine biosynthesis and catabolism A number of genes (MYC, P53, KRAS, BRAF and YAP/TAZ) are involved in the regulation of intracellular polyamine pools. KLF6, Kruppel-like factor 6; PPARγ, peroxisome activated receptor γ; uPAR, urokinase-type plasminogen activator receptor; Cav-1, caveolin-1; CPS1, carbamoyl phosphate synthetase 1; OTC, ornithine transcarbamylase; ARG1, arginase-1; ODC1, ornithine decarboxylase; OAT, ornithine aminotransferase; SSAT, spermidine/spermine N (1)-acetyltransferase 1; SRM, spermidine synthase (Created in BioRender. Liu, H. (2025) https://BioRender.com/y76e444).

Effective control of polyamine uptake and biosynthesis has been proposed as an effective anticancer strategy, given the crucial role of polyamines in cell proliferation. Several oncogenes and tumor suppressor genes are involved in the regulation of intracellular polyamine levels and contribute to the continued proliferation of cancer cells ( Figure 2).

#### MYC

The interaction between oncogenes and polyamine metabolism is demonstrated by the direct activation of ODC1 by the *MYC* gene, thereby stimulating the biosynthesis of the polyamines required for cell division and proliferation [28]. Several types of cancers lead to significant increases in MYC expression through gene duplication or mutation. This upregulation of MYC, in turn, controls polyamine biosynthesis, maintaining high levels of intracellular polyamines and promoting cancer cell survival [ 29– 35]. Increased levels of polyamines contribute to the development of tumors by enhancing the deoxyhypusine synthase (DHPS)-mediated hypusination of the translation factor eIF5A, which in turn stimulates the production of MYC [36]. In colorectal cancer patients, the concurrent inhibition of ODC and eIF5A led to the suppression of MYC in tumor cells, demonstrating a synergistic antitumor effect [36]. Furthermore, SMS is an essential enzyme for colon cancer cells to metabolize excess spermidine, and combined inhibition of SMS and MYC expression has synergistic antitumor effects, inducing apoptosis and inhibiting tumor growth [37]. Polyamines and their precursors, such as arginine, play a significant role in enhancing cancer cell sensitivity to ferroptosis via the WNT/MYC signaling pathway. ODC1 is a transcriptional target of MYC downstream of the WNT pathway, which increases the biosynthesis of PAs, leading to their accumulation in the TME. This accumulation plays a crucial role in modulating the susceptibility of cells to ferroptosis signals [38].

#### KRAS

Mutations in KRAS hyperactivate downstream signaling pathways that drive cancer initiation [39]. KRAS mutations are among the most common and challenging mutations, with up to 95% of pancreatic ductal adenocarcinomas harboring KRAS mutations. They play a critical role in the metabolic plasticity and rewiring of pancreatic ductal adenocarcinoma cells [40]. Recent studies demonstrated that the KRAS (G12D) mutant is able to induce ornithine transaminase (OAT) and ODC1 to support the biosynthesis of polyamines, which are essential for tumor proliferation and thereby promote tumor growth [ 16, 41]. *KRAS* is also one of the most frequently mutated oncogenes in colorectal cancer; it is carried by approximately 40% of patients with colorectal cancer and is associated with a poor prognosis. Activated KRAS leads to increased interactions between uPA/uPAR and subsequent SRC activation by upregulating uPAR expression, which enhances the phosphorylation of caveolin-1 (Cav-1) tyrosine residues and promotes the internalization of polyamines through endocytosis [42]. Furthermore, KRAS inhibits the expression of SSAT by disrupting the transcriptional activation of peroxisome proliferator-activated receptor gamma (PPARγ), thereby leading to the maintenance of elevated levels of polyamines in colorectal cancer cells [43]. The activation of PPARγ is positively correlated with an increase in SSAT in colorectal cancer cells [44]. However, the development of KRAS-mutant suppressor molecules has not been successful.

#### BRAF

Mutations in the *BRAF* gene are detected in half of all melanoma tumors, and 90% of BRAF mutations result in the substitution of valine at amino acid 600 for glutamic acid (V600E mutation) [45]. BRAF mutations drive constitutive activation of the RAS-RAF-MEK-ERK signaling pathway, promoting uncontrolled cell proliferation [46]. Mutant BRAF melanomas are more dependent on polyamine biosynthesis and upregulated polyamine transport systems to maintain high intracellular polyamine levels and exhibit increased sensitivity to targeted polyamine transport system inhibitors [47]. These findings provide a basis for the potential use of polyamine-like compounds in treatment, which may reduce the occurrence of resistance to BRAF inhibitors.

#### P53

*TP53* gene encodes a transcription factor for the p53 tumor suppressor protein, which plays a crucial role in controlling cell division, apoptosis, and DNA repair. Mutations or loss of the *TP53* gene can lead to uncontrolled growth and division of damaged cells, promoting cancer progression. During the metabolism of colorectal tumor cells, TP53 downregulates the expressions of key enzyme-encoding genes of the urea cycle, including carbamoyl phosphate synthetase 1, ornithine transcarbamylase, and arginase-1, reducing urea production and ammonia consumption, thereby inhibiting tumor growth [48]. After the loss of TP53 in colon tumor cells, ammonia accumulation increases ODC1 transcription in tumor cells, ultimately leading to increased intracellular polyamine biosynthesis [48]. During colorectal adenoma development, the host’s urea cycle metabolism is markedly activated. Urea infiltrates macrophages, disrupting the binding of p-STAT1 to the *SAT1* promoter region, consequently resulting in the accumulation of polyamines within macrophages [49].

#### JUN and FOS

*JUN* and *FOS* are two nuclear oncogenes that form the AP-1 complex when they combine. This complex binds to DNA, promoting cell proliferation, transformation, and carcinogenesis, and is closely associated with the metastasis of tumor cells. In the promoter regions of polyamine metabolism-related genes, such as the *ODC* gene promoter, there are AP-1 binding sites. c-JUN and c-FOS activate the transcription of downstream genes by binding to these sites. Research has shown that overexpression of ODC in colorectal cancer cells can significantly increase the mRNA levels of *c-JUN* and *c-FOS*, indicating that ODC may activate the transcription of *c-JUN* and *c-FOS* through several mechanisms, thereby promoting their expressions [50]. These findings suggest that JUN and FOS are involved in stimulating polyamine biosynthesis, thereby promoting tumor growth. In polyamine metabolism, the expressions of c-JUN and c-FOS are influenced by polyamine analogues. For example, in human breast cancer MDA-MB-435 cells, oligoamine polyamine analogues can significantly induce the expressions of c-JUN and c-FOS, as well as the phosphorylation of c-JUN, suggesting that c-JUN and c-FOS may be involved in the cellular response process induced by polyamine analogues [51]. Inhibiting the activity of c-JUN and c-FOS or their downstream signaling pathways may enhance the cytotoxic effects of polyamine analogues on tumor cells, thereby improving their efficacy in cancer treatment.

#### YAP/TAZ

Aberrant activation of YAP/TAZ is associated with invasive phenotypes and poor prognosis and has been identified in various cancers, such as breast cancer, ovarian cancer, glioblastoma, and liver cancer [52]. YAP/TAZ directly upregulates the transcription of *ODC1*, increasing polyamine levels within cancer cells and promoting cancer cell proliferation and tumor growth [53]. In cancer datasets, the upregulation of ODC1 is positively correlated with the mRNA levels of *YAP*/ *TAZ*. By binding to the TEA domain, YAP/TAZ promotes the transcription of *ODC1*, and decreasing the expression of ODC1 reduces the proliferation of liver cancer cells. An increase in polyamine levels leads to the overexpression of eIF5A, promoting the translation of the transcriptional repressor lysine-specific demethylase 1 (LSD1). LSD1 mediates the activation of many tumor suppressor genes or negative regulators of cell proliferation by YAP/TAZ, which act as transcriptional repressors.

### Signaling pathway and polyamine metabolism

**Figure 3:**
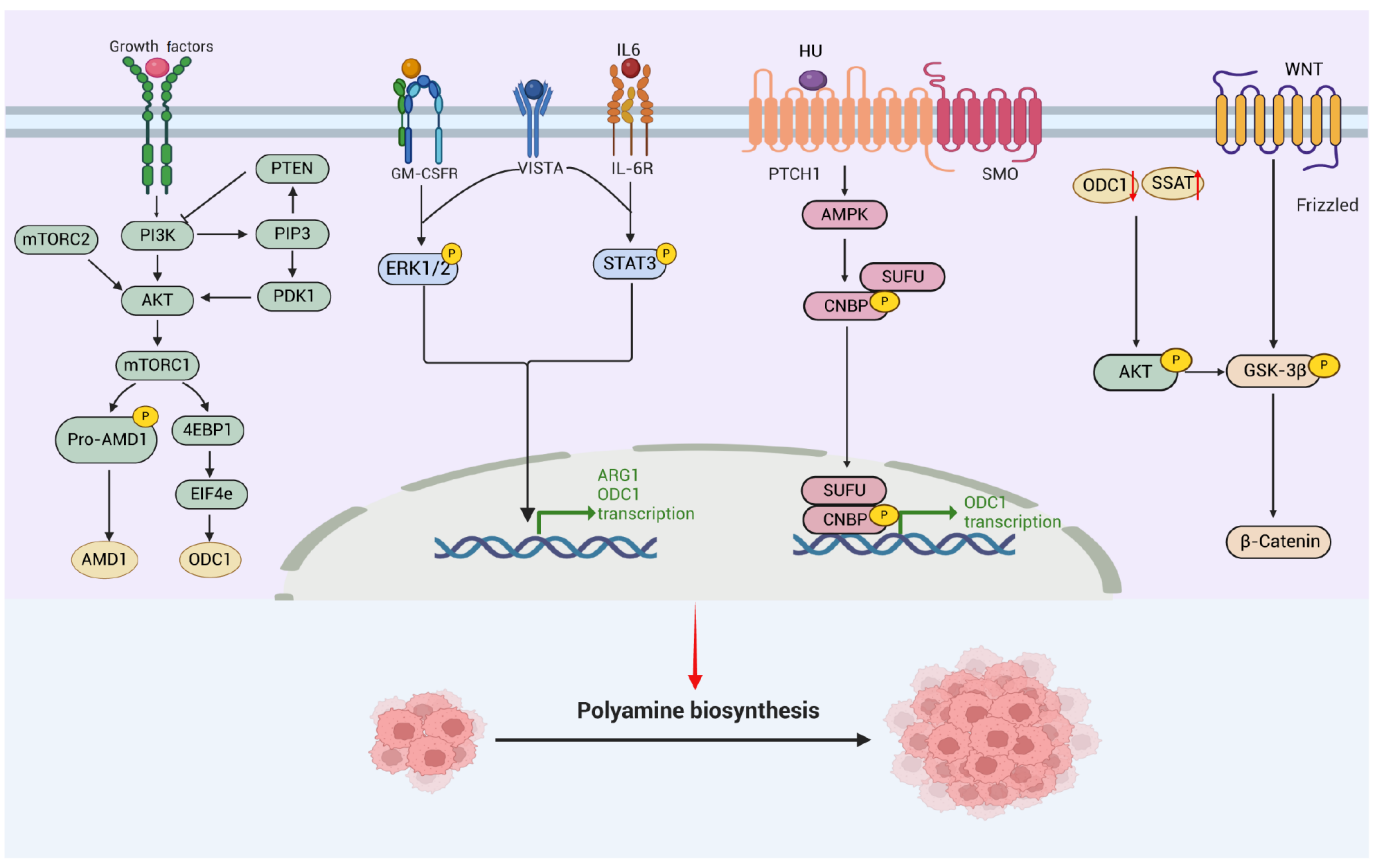
Figure 3 Major signaling pathways and underlying factors affecting polyamine biosynthesis The major signaling pathways of the PI3K/AKT/mTOR, WNT/β-catenin, Hedgehog, ERK and STAT3 pathways, as well as their regulatory roles in polyamine biosynthesis. MEK, mitogen-activated protein kinase kinase; ERK1/2, extracellular signal-related kinase 1/2; PI3K, phosphoinositide 3-kinase; AKT, protein kinase B; mTORC1/2, mammalian target of rapamycin complex 1/2; PTEN, phosphatase and tensin homologue; PDK1, phosphoinositide-dependent protein kinase 1; 4E-BP1, eukaryotic translation initiation factor 4E (eIF4E)-binding protein 1; STAT3, signal transducer and activator of transcription 3; AMPK, AMP-activated protein kinase; LSD1, lysine-specific demethylase 1; GSK3β, glycogen synthase kinase 3; CNBP, CCHC type nucleic acid binding protein (Created in BioRender. Liu, H. (2025) https://BioRender.com/x95n202).

Polyamine metabolism can be regulated by multiple signaling pathways, including the PI3K/AKT/mTOR, WNT/β-catenin, Hedgehog, ERK [54] and STAT3 [54] signaling pathways, revealing the extensive communication between polyamines and multiple signaling pathways ( Figure 3).

#### PI3K/AKT/mTOR signaling pathway

mTOR is a protein kinase that regulates cell proliferation and metabolism and is activated by the combined action of growth factors, energy, and amino acids. The activation of the PTEN-PI3K-mTORC1 pathway is an important metabolic mechanism that maintains cancer cell growth and proliferation. Through the activation of this pathway, the metabolic capacity of cancer cells is increased to support the nutrition required for cancer cell proliferation and spread. A broad interplay between polyamines and mTOR signaling is beginning to emerge, with activated mTORC1 producing large amounts of polyamines by upregulating AMD1 in human prostate cancer [55]. Inhibition of mTOR leads to decreased activity of AMD1 and reduced intracellular polyamine levels. The translation of ODC is associated with EIF4E activity, which is controlled by the mTORC1-4EBP axis, potentially promoting the conversion of ornithine to putrescine [ 56, 57]. Furthermore, mTORC1 has been shown to stabilize the association of the mRNA-binding protein HuR (also known as ELAVL1) with *ODC* transcription, stabilizing *ODC* mRNA in RAS-transformed cells [58]. In addition, macrophages produce large amounts of spermidine and spermine through the mTORC1 signaling pathway [58]. These polyamines are absorbed by epithelial cells, leading to a metabolic shift in the cells, promoting their proliferation and enhancing their defense mechanisms. To date, it has not been determined whether mTOR inhibition can synergistically reduce tumor growth in conjunction with the inhibition of polyamine biosynthesis.

#### Hedgehog signaling pathway

Previous studies have implicated Hedgehog signaling in the upregulation of polyamine biosynthesis in cerebellar granule cell precursors of medulloblastoma [59]. The protein levels of ODC and polyamine biosynthesis are increased downstream of the Hedgehog signaling pathway through the activation of 5′-AMP-activated protein kinase (AMPK) [59]. The noncanonical Hedgehog signal activates AMPK to phosphorylate CCHC-type zinc finger nucleic acid-binding protein (CNBP), leading to increased translation of ODC and polyamine biosynthesis, thereby inhibiting the growth of primary medulloblastoma cells in a mouse xenograft model.

#### Wnt/β-catenin signaling pathway

Research has shown that in human Hepatocellular carcinoma and colon cancer cell models, the polyamine depletion caused by SSAT overexpression is a result of decreased AKT signaling and reduced nuclear β-catenin, leading to decreased tumor cell growth, migration, and invasion [60]. Excessive SMS reduces the accumulation of spermidine by converting it into spermine. Spermidine activates the serine/threonine kinase and epithelial-mesenchymal transition signaling pathways, thereby inhibiting the proliferation and invasion of pancreatic cancer cells [61].

## Anticancer Strategies Targeting Polyamine Metabolism

### Blocking polyamine biosynthesis and polyamine transport systems

Polyamine metabolic pathways have been validated as rational targets for therapeutic intervention, leading to the development of a variety of inhibitors that target polyamine metabolic enzymes and polyamine transport systems. These inhibitors have shown efficacy in cancer prevention and treatment in animal models, with some already advancing to clinical trial stages.

ODC is a key enzyme in polyamine biosynthesis, and its inhibitor, difluoromethylornithine (DFMO), is a small-molecule drug that was originally developed as a potential anticancer agent in the 1970s. The most successful inhibitor designed against polyamine biosynthetic enzymes to date is DFMO, which irreversibly inhibits ODC, leading to depletion of intracellular polyamines and thereby suppressing cell proliferation [ 6, 14]. In the treatment of cancers such as skin cancer, breast cancer, and colon cancer, DFMO has been tested as a monotherapy in clinical trials, but its single-agent therapeutic effect is not significant [ 62– 65]. It was serendipitously found to be effective against human African trypanosomiasis, commonly known as sleeping sickness, and was approved by the U.S. Food and Drug Administration (US FDA) in 1990 [ 66, 67]. Recently, several studies have shown that taking DFMO orally for two years after immunotherapy for high-risk neuroblastoma can effectively reduce long-term recurrence, and it has been officially approved by the US FDA for the treatment of high-risk neuroblastoma [ 68– 70]. In addition, the combination therapy of DFMO with the polyamine transport system has made some progress in cancer treatment. AMXT 1501 and Trimer44NMe are now recognized as polyamine transport system inhibitors (PTIs), and their combination with DFMO can deplete the intracellular polyamine pool in multiple cancer types, thereby inhibiting tumor growth and antitumor immunity and improving survival in mice [ 71– 74]. A phase 1B/2A clinical trial of a combination of AMXT 1501 and DFMO in patients with cancer is currently underway (NCT05500508).

In addition, researchers have developed compounds similar to natural polyamine substances that ideally reduce the concentration of intracellular polyamines and reduce their proliferative capacity in tumor cells. For example, the spermine analogue diethyldihydroxyhomospermine (SBP-101) has demonstrated efficacy in slowing the progression of pancreatic tumors both *in vitro* and *in vivo* and has now entered phase II/III clinical trials (NCT05254171) [75]. *N*11-bis(ethyl) norspermine (BENSpm) can induce the polyamine catabolic enzymes SSAT and SMOX, leading to rapid depletion of polyamines and an increase in reactive oxygen species (ROS), resulting in antitumor activity and enhancing the antitumor effect of chemotherapeutic drugs to a certain extent [ 76, 77]. Like BENSpm, PG-11047 depletes intracellular polyamine pools by inhibiting polyamine biosynthesis, resulting in the inhibition of tumor growth in various types of cancers and the ability of PG-11047 to enter clinical trials for advanced cancer patients [ 78, 79]. However, the role of these inhibitors in clinical cancer treatment needs to be further verified. Details of the development of compounds that affect polyamine metabolism have been covered elsewhere [ 8, 14].

### Antitumor strategies involving polyamine supplementation *in vitro*

**Figure 4:**
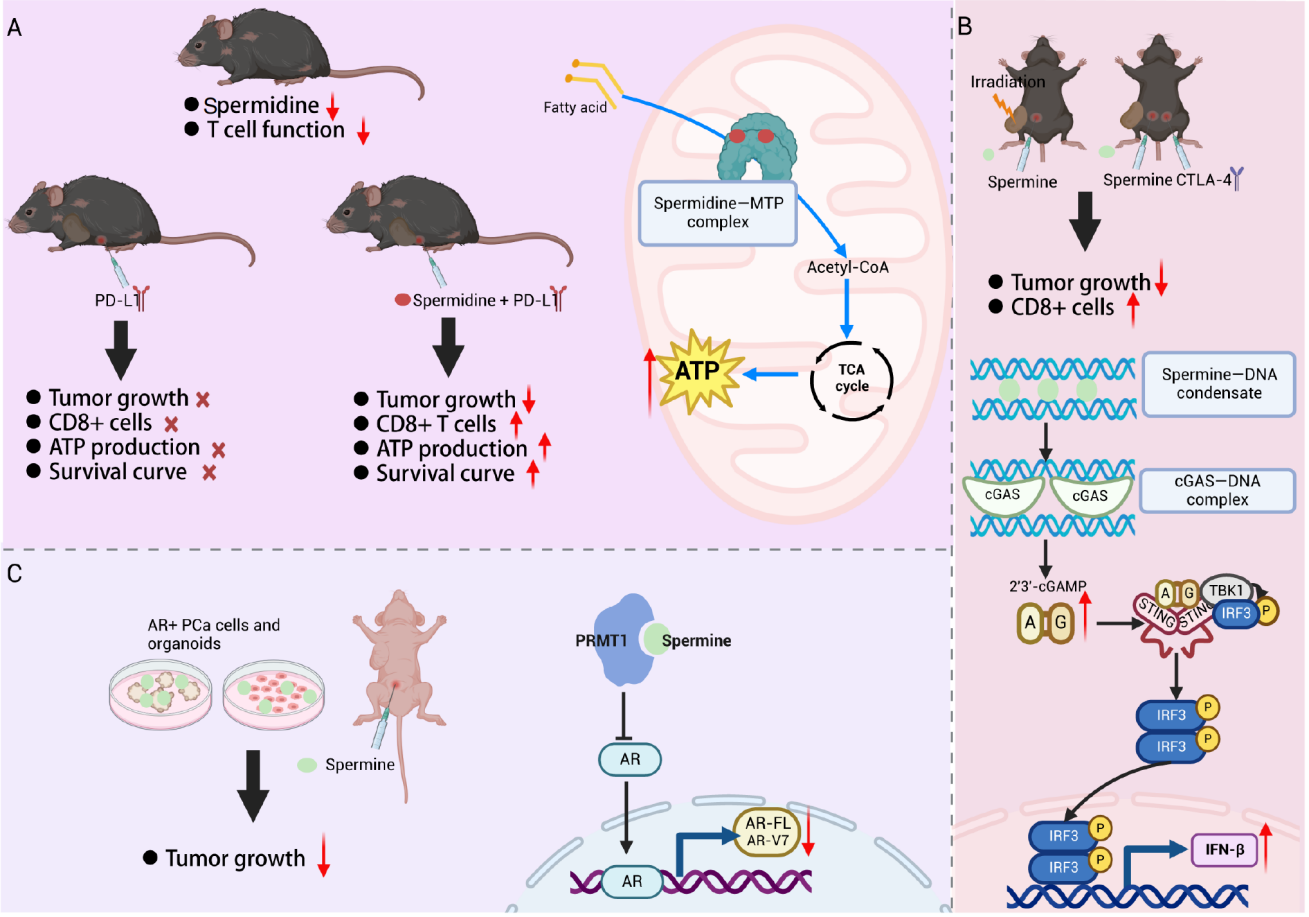
Figure 4 Antitumor strategies involving polyamine supplementation *in vitro* (A) Spermidine supplementation can increase fatty acid oxidation activity and improve the mitochondrial activity of CD8+ T cells in aged mice to enhance the antitumor immune response. (B) Spermine enhances antitumor effects by stabilizing DNA binding to cGAS and promoting the cGAS-STING signaling pathway. (C) Spermine can significantly downregulate the AR-FL and AR-V7 signaling pathways by targeting the protein arginine methyltransferase PRMT1 and plays an antitumor role in CRPC. CTLA-4, Cytotoxic T-lymphocyte antigen 4; PD-L1, programmed cell death ligand 1; cGAS, cyclic GMP-AMP synthase; STING, stimulator of the interferon gene; TBK1, TANK-binding kinase 1; IRF3, interferon regulatory factor 3; 2′3′-cGAMP, 2′3′-cyclic GMP-AMP; MTP, microsomal triglyceride transfer protein; ATP, adenosine triphosphate; AR, androgen receptor; AR-FL, full-length androgen receptor; AR-V7, androgen receptor splice variant 7; PRMT1, protein arginine methyltransferase; INF-β, interferon beta (Created in BioRender. Liu, H. (2025) https://BioRender.com/j72c071).

Spermidine is the central hub of polyamine metabolism and plays an important role in cell growth, development, protein/nucleic acid synthesis, and cell signaling. The spermidine level decreases with age in the human body, and supplementation with spermidine can improve or delay age-related diseases. Spermidine plays a “multifaceted” role in anti-aging, antitumor, immune regulation, cardiovascular protection, neuroprotection, support of stem cells, *etc*., demonstrating enormous potential [15]. In addition, spermidine supplementation has been shown to enhance antitumor immune responses in animal models. Researchers have shown that spermidine supplementation enhances the activity and function of mitochondria in elderly CD8+ T cells by directly binding to and activating the mitochondrial trifunctional protein (MTP) to promote fatty acid oxidation, thereby improving the antitumor effect of PD-1 immunosuppressive therapy [80] ( Figure 4A). However, it is worth noting that spermidine itself does not have antitumor effects. Studies have shown that spermidine, an endogenous polyamine released by tumor cells in the microenvironment, inhibits T-cell antigen receptor aggregation by downregulating plasma membrane cholesterol levels, thereby inhibiting T-cell activation [80].

Cyclic GMP-AMP synthetase (cGAS) mediates protective immune responses to microbial infections and is involved in a variety of noninfectious diseases, such as inflammatory diseases, antitumor immunity, and cellular senescence [81]. Zhao *et al*. [82] demonstrated that spermine and spermidine induce B-to-Z DNA transformation to inhibit dsDNA binding to cGAS and that the polyamine catabolic enzyme SAT1 promotes cGAS activation by inhibiting cellular Z-DNA accumulation. In addition, spermine enhances and stabilizes the binding of cGAS to DNA by polymerizing the DNA, which further activates downstream cGAS-STING signaling to enhance antiviral and antitumor effects [83] ( Figure 4B). In addition, the level of spermine in prostate cancer tissues is lower than that in adjacent tissues [84]. Moreover, the degree of malignancy of prostate cancer is negatively correlated with the level of spermine [85]. The accumulation of intracellular spermine inhibits tumor cell proliferation by directly binding to its endogenous target, protein arginine methyltransferase 1 (PRMT1), to suppress the transcription of the androgen receptor (AR) and thereby inhibit tumor growth [86] ( Figure 4C). In addition, polyamine supplementation can increase the sensitivity of cancer cells or xenograft tumors to radiotherapy or chemotherapy by inducing ferroptosis [38].

These studies suggest that whether polyamines that are abnormally changed during cancer progression intervene in tumor progression is poorly understood. It is necessary to further explore the endogenous physiological mechanism by which polyamines affect tumor development and to provide new ideas for the development of polyamines that cooperate with downstream targets or immunotherapy to block tumor growth.

## Conclusions and Future Directions

As our understanding of the pathways affected by polyamines and the molecular mechanisms involved in polyamines develops, the potential to exploit polyamine metabolic pathways as a strategy for cancer treatment and prevention has increased. The discovery that polyamines are directly involved in tumor immune responses as well as tumor growth or through multiple oncogenic signaling pathways also provides new points of intervention. However, many drugs targeting polyamine metabolism have been developed but have not achieved good results in clinical trials. In addition, with increasing global age, the incidence of cancer is expected to increase, so early screening and comprehensive treatment for cancer are urgently needed.

The fields of polyamine metabolism and cancer hold considerable potential for future research and development. Fundamentally, a deeper investigation into the role of polyamine metabolism within the tumor microenvironment is warranted. This includes studying its impact on processes such as tumor angiogenesis, immune evasion, and remodeling of the extracellular matrix, as well as understanding how the tumor microenvironment in turn influences polyamine metabolism. Additionally, there is a need to explore the interplay and regulatory mechanisms between polyamine metabolism and other key metabolic pathways, including glycolysis, lipid metabolism, and amino acid metabolism. Research should also focus on the heterogeneity of polyamine metabolism across various types of tumors. For clinical application, it is essential to design and develop novel inhibitors of polyamine metabolism with increased specificity, efficacy, and safety. Investigating the synergistic effects of combining polyamine metabolism inhibitors with other anticancer agents and refining the dosing and timing of combination therapies could yield significant benefits. Further validation of polyamines and their metabolites as biomarkers for tumor diagnosis and prognosis is necessary, with the potential for developing liquid biopsy technologies based on polyamine metabolism. The role of polyamine metabolism in tumor immune evasion should be examined to identify strategies for enhancing the effectiveness of tumor immunotherapy through the modulation of polyamine metabolism. Additionally, studying the involvement of polyamine metabolism in tumor metabolic reprogramming and its implications for overcoming drug resistance in cancer is highly important. Overall, the exploration of polyamine metabolism in relation to cancer is a promising area of study. It has the potential to advance our understanding of cancer metabolism and to contribute to the development of innovative therapeutic strategies for cancer treatment.
